# Lack of Macrolide Resistance in *Chlamydia trachomatis* after Mass Azithromycin Distributions for Trachoma

**DOI:** 10.3201/eid1507.081563

**Published:** 2009-07

**Authors:** Kevin Cyrus Hong, Julius Schachter, Jeanne Moncada, Zhaoxia Zhou, Jenafir House, Thomas M. Lietman

**Affiliations:** University of California, San Francisco, California, USA

**Keywords:** Trachoma, chlamydia, azithromycin, doxycycline, macrolides, antimicrobial resistance, Ethiopia, dispatch

## Abstract

We investigated antimicrobial drug resistance in ocular *Chlamydia trachomatis* 18 months after 4 biannual communitywide distributions of antimicrobial drugs in a region of Ethiopia where ocular strains of *C. trachomatis* are highly endemic. We found no significant differences in susceptibilities to azithromycin and doxycycline in 6 posttreatment and 4 pretreatment samples.

Trachoma, a sequela of repeated conjunctival infection with *Chlamydia trachomatis*, is the leading cause of infectious blindness (*1*). To control endemic trachoma, the World Health Organization (WHO) recommends communitywide distribution of antimicrobial agents, along with surgery and improved hygiene. Mass azithromycin treatments have been effective in reducing this infection (*2*). However, concerns have been raised that selective antimicrobial pressure may produce macrolide-resistant strains of *C. trachomatis* and other pathogens (*3*).

Solomon et al. reported on antimicrobial drug susceptibility in 9 chlamydial isolates 2 months after mass antimicrobial drug treatment in Tanzania (*4*). These authors observed a slight increase in the median MIC after treatment but found no resistant strains. Despite this encouraging study, investigations of the long-term impact of multiple treatments on antimicrobial drug susceptibility are needed. Geographic areas where trachoma is hyperendemic require repeated mass distributions because infection has been shown to return after a single treatment (*5*). Antimicrobial drug–resistant *C. trachomatis* might not emerge until multiple treatments have occurred. For example, in Nepal, azithromycin-resistant pneumococcal strains were observed only after consecutive annual treatments (*3*). The selective pressure of repeated azithromycin distributions might enable rapid expansion of resistant clones (*6*). In 2006, we investigated antimicrobial drug resistance in ocular *C. trachomatis* 18 months after 4 biannual treatments (2003–2004) in a trachoma-endemic region of Ethiopia.

## The Study

We obtained ethical approval from the Committee on Human Research at the University of California, San Francisco (UCSF), and from the National Ethical Clearance Committee of the Ethiopian Science and Technology Agency. Antimicrobial treatments were distributed every 6 months to 24 randomly selected villages in the Gurage zone in Ethiopia. Persons >1 year of age were offered single-dose oral azithromycin (1 g for adults or 20 mg/kg for children) as directly observed treatment. Pregnant women and those allergic to macrolides were offered a 6-week course of topical 1% tetracycline ointment (applied 2×/day to both eyes, not directly observed). At each biannual visit, 8 villages not yet receiving antimicrobial drug distribution were randomly selected from the same district to serve as controls (*5*). The same procedures were conducted in the control villages as in the treatment villages. After completion of clinical ocular examinations and sample collection, treatment was offered to study and control villages.

All children 1–5 years of age received ocular examinations, and conjunctival swabs were taken from their right tarsal conjunctiva before treatment. Swabs were placed immediately in M4RT media (Remel, Lenexa, KS, USA) at 4°C, frozen at –20°C within 6 hours, and transported at 4°C to the microbiology laboratory at the Proctor Foundation at UCSF, where they were stored at –80°C until PCR was performed. An aliquot of each sample was processed with the Amplicor PCR test (Roche Molecular Systems, Branchburg, NJ, USA) for detection of *C. trachomatis*. At 18 months after the fourth biannual treatment, the prevalence of ocular infection in preschool children was 10.7% (95% confidence interval [CI] 5.5%–19.3%) (*7*). In control villages that had not yet received treatment, the average prevalence was 31.2% (95% CI 23.1%–40.5%). Of 552 samples from 8 biannually treated villages, 59 were positive by PCR. Of 523 samples from 8 control villages, 163 were PCR positive. Among these PCR-positive samples, 10 were randomly chosen from biannually treated villages and 10 from control villages. Remnants of the original specimens were sent to the UCSF Chlamydia Laboratory for culture and antimicrobial drug susceptibility testing.

Technicians were unaware of the origin of each sample, and the order in which samples were tested was randomized. *C. trachomatis* isolation was carried out in cycloheximide-treated McCoy cells in 1-dram shell vials by using a modification of the procedure of Ripa and Mardh (*8*). We sequenced the *ompA* gene from all conjunctival isolates (*9*). The protocol for determining antimicrobial drug sensitivity (*10*) was used with minor modifications. Azithromycin (American Pharmaceutical Partners, Schaumburg, IL, USA) and doxycycline (Bedford Laboratories, Bedford, OH, USA) were tested against chlamydia isolates; MICs were conducted in triplicate, and minimum chlamydicidal concentrations (MCCs) in duplicate. Each MIC or MCC included a clinical chlamydia isolate with a known susceptibility to azithromycin and doxycycline as a positive control to ensure the validity of the sensitivity testing.

Among 10 Amplicor PCR–positive samples randomly chosen from biannually treated villages, cultures for 7 samples were positive; 6 of these were tested for resistance against azithromycin and doxycycline (Table). Of 10 random Amplicor PCR–positive samples from pretreatment villages, cultures for 6 were positive; 4 of these samples were tested for resistance (Table).

**Table:** Antimicrobial drug susceptibilities of *Chlamydia trachomatis*, Ethiopia*

Treatment status	Serotype	Azithromycin†			Doxycycline†	
		MIC	MCC		MIC	MCC
4 biannual	A/Har13	0.5	0.5		0.03	0.03
	Ba/Apache-2	0.5	0.5		0.03	0.03
	Ba/Apache-2	0.5	0.5		0.03	0.06
	Ba/Apache-2	0.25	0.5		0.03	0.06
	Ba/Apache-2	0.5	0.5		0.03	0.06
	Ba/Apache-2	0.5	0.5		0.03	0.03
None	A/Har 13	0.5	0.5		0.03	0.03
	A/Har 13	0.5	0.5		0.03	0.03
	A/Har 13	0.5	1		0.015	0.06
	Ba/Apache-2	0.25	0.25		0.03	0.03

*Samples taken in 2006, 18 months after 4 biannual treatments in 2003–2004. MCC, minimum chlamydicidal concentration. †MIC and MCC values given in μg/mL.

MICs and MCCs were comparable between biannually treated villages and control villages for the 2 antimicrobial drugs tested. No statistically significant differences were found (p = 0.76 for azithromycin MIC; p = 1.00 for azithromycin MCC; p = 0.22 for doxycycline MIC; and p = 0.45 for doxycycline MCC) (Wilcoxon rank-sum test, STATA, StataCorp, College Station, TX, USA). With the number of samples tested (6 posttreatment samples and 4 pretreatment samples), we had 95% power to detect a 2-fold shift in the mean MIC (α = 0.05, two-sided) and 90% power to detect a 2-fold shift in the mean MCC of azithromycin. For doxycycline, we had 99% power to detect a 2-fold change in the mean MIC (α = 0.05, two-sided) and 85% power to detect a 2-fold change in the mean MCC.

## Conclusions

This study found no significant increase in antimicrobial drug resistance against azithromycin or doxycycline after treatment for ocular *C. trachomatis*. Our study had limited power to detect a rare mutation that can engender resistant chlamydia strains but sufficient power to detect a major shift in antimicrobial drug susceptibilities. In a region like Ethiopia, where trachoma is endemic, infection often returns to the community after mass distribution of antimicrobial drugs stops (*7*). Concerns exist that resistant strains may result in failure to eliminate trachoma locally, but our study refutes this concern. The lack of antimicrobial drug resistance shown in this study is particularly encouraging because the region was subjected to higher selective pressures from biannual treatments than would be expected from annual treatments recommended by WHO.

Chlamydiae are obligate intracellular bacteria that can multiply only in the cytoplasm of a susceptible host cell, and acquisition of antimicrobial drug resistance genes from other organisms through horizontal transfer is probably rare. In vitro spontaneous mutations in chlamydia can confer antimicrobial drug resistance, including that in the 23S rRNA, but mutations appear to incur competitive disadvantages (*11*).

The persistent pressure of repeated mass treatments may select for compensatory mutations that could adapt chlamydia to the fitness cost of the initial mutation (*11*). This scenario would enable the expansion of resistant strains after communitywide distribution of azithromycin in trachoma control programs. However, after treatment is discontinued, sensitive wild type strains may outcompete resistant ones. In one Australian community, the increased population of azithromycin-resistant pneumococcus was quickly replaced by sensitive strains after treatment ceased (*6*). In our study, if resistant strains emerged after 4 biannual antimicrobial drug treatments, they did not survive the competition with sensitive strains 18 months after mass treatment stopped.

Determining antimicrobial drug resistance in *C. trachomatis* is technically challenging, has no standardized protocol (*12*), and is rarely pursued. DNA sequencing to identify genetic mutations that confer macrolide resistance could be more sensitive but would require further efforts to characterize specific mutations causing macrolide resistance. Mass distribution of antimicrobial drugs, especially azithromycin, is a critical component in the global elimination of blinding trachoma. Emergence of antimicrobial drug resistance to chlamydia treatments would be detrimental to the goal of eliminating trachoma, so surveillance for resistance should be part of trachoma programs.
